# Improving prediction of burial state of residues by exploiting correlation among residues

**DOI:** 10.1186/s12859-017-1475-5

**Published:** 2017-03-14

**Authors:** Hai’e Gong, Haicang Zhang, Jianwei Zhu, Chao Wang, Shiwei Sun, Wei-Mou Zheng, Dongbo Bu

**Affiliations:** 1 Key Lab of Intelligent Processing, Institute of Computing Technology, Chinese Academy of Sciences, Beijing, 100190 China; 2 School of Computer Science, University of Chinese Academy of Sciences, Beijing, China; 3 Institute of Theoretical Physics, Chinese Academy of Sciences, Beijing, 100190 China

**Keywords:** Protein structure, Burial states of residue, Conditional random field, Residue correlation

## Abstract

**Background:**

Residues in a protein might be buried inside or exposed to the solvent surrounding the protein. The buried residues usually form hydrophobic cores to maintain the structural integrity of proteins while the exposed residues are tightly related to protein functions. Thus, the accurate prediction of solvent accessibility of residues will greatly facilitate our understanding of both structure and functionalities of proteins. Most of the state-of-the-art prediction approaches consider the burial state of each residue independently, thus neglecting the correlations among residues.

**Results:**

In this study, we present a high-order conditional random field model that considers burial states of all residues in a protein simultaneously. Our approach exploits not only the correlation among adjacent residues but also the correlation among long-range residues. Experimental results showed that by exploiting the correlation among residues, our approach outperformed the state-of-the-art approaches in prediction accuracy. In-depth case studies also showed that by using the high-order statistical model, the errors committed by the bidirectional recurrent neural network and chain conditional random field models were successfully corrected.

**Conclusions:**

Our methods enable the accurate prediction of residue burial states, which should greatly facilitate protein structure prediction and evaluation.

## Background

According to their solvent accessible area, protein residues can be categorized into two classes, i.e., buried and exposed [1]. Buried residues commonly form hydrophobic cores, maintaining the conformation and structural integrity of proteins. In contrast, exposed residues tend to appear on the surface of proteins and partly determine protein functions through interactions with other proteins or ligands. Thus, the solvent accessibility of residues is one of the driving forces of protein folding. In addition, solvent accessibility is an important global feature of residues that is complementary to the other local features; it can also be easily predicted compared with other global features such as contact map [2–4].

An accurate prediction of solvent accessibility can provide important structural information for the study of protein evolution, structure, and function [5]. Most of the existing prediction approaches employ the following strategy. First, a fixed-length window is opened around the residue of interest and a feature vector is computed based on the sequence information within this window. The most widely-used features include residue types [6], position specific scoring matrix (PSSM) [7–9], and predicted secondary structure (SS) [10]. In addition, the real solvent accessibility of the residue of interest is computed using the dictionary of protein secondary structure (DSSP) as burial state labels [11]. Second, these feature vectors along with burial state labels are inputted into a machine learning model such as artificial neural network (ANN) [5, 8, 12–21], support vector machine (SVM) [9, 10, 20, 22–24], deep learning model [25], conditional neural field (CNF) [2], and random forest (RF) [6] for training. Finally, the trained model is used for predicting solvent accessibility of protein residue in a testing set. Among these approaches, bidirectional recurrent neural network (BRNN) shows excellent performance and has been widely used in softwares such as SCRATCH [26] and ACCpro [5].

**Fig. 1:**
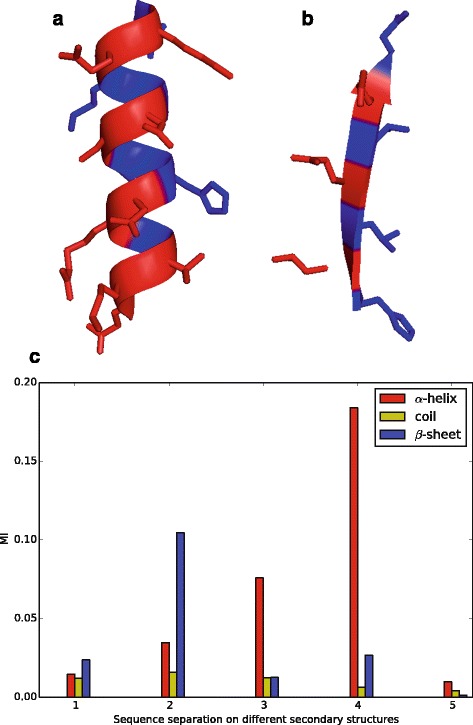
Correlation among burial states of residues. In panel (**a**) and (**b**), buried residues are shown in *blue*, while exposed residues are shown in *red*. In panel (**c**), mutual information (MI) of burial states is calculated to measure the correlation among residue pairs. These figures clearly show the strong correlation of burial states among residues. **a** Periodicity of burial states of residues on *α*-helices. **b** Periodicity of burial states of residues on *β*-strands. **c** Correlation of burial states of residue pairs as function of sequence separation between these residues

These prediction approaches have shown success; however, most of these approaches consider the residue of interest independently and thus, neglect the correlations among residues. In fact, the burial state of residues presents strong correlation. As shown in Fig. 1, two residues with a sequence separation of 3 or 4 amino acids in *α* helices tend to adopt identical burial states due to local geometry preference, and two residues with a sequence separation of 2 amino acids in *β* strands commonly take identical burial states under the effect of hydrogen bonds. In contrast, the residue pairs on coils show relatively weak correlation. Thus, the incorporation of these correlations, including correlations among adjacent residues and long-distance residues, into the prediction model remains a challenge.

In this study, we present a high-order conditional random field (CRF) model to explicitly exploit the correlations among all residues rather than consider each residue individually. This statistical model includes a collection of doublet terms to describe correlations among adjacent residues and long-distance residue pairs as well. To investigate the effect of correlations, we compared our approach with the state-of-the-art models that neglect correlations. In addition, we also investigated the effect of different features for prediction accuracy. Experimental results on two benchmark datasets showed that our approach has higher accuracy than the existing methods.

## Results and discussion

### Datasets

We tested our approach on two benchmark datasets, i.e., i) training and validation data collected from SCOP70 and ii) independent testing data collected from PDB25. A filtering pre-processing was performed to guarantee no overlapping between these two datasets.

#### Training and validation dataset

The training dataset was constructed based on SCOP70 with filtering procedure. In particular, the proteins with chain-breaks or less than 50 residues were excluded. Besides, membrane proteins were also excluded. As a result, a total of 2349 proteins, including 505 *α* proteins, 552 *β* dataset, 706 *α*/*β*, and 586 *α*+*β*, were obtained after filtering. Five-fold cross-validation (5-CV) was used for our evaluation, i.e., these proteins were randomly divided into five subsets with equal size: four subsets were selected as training set, and one subset was selected as validation dataset.

#### Independent testing dataset

The testing data was constructed based on PDB25. To avoid overlap with the training data set, only newly-released proteins were selected (released after Aug. 1st, 2015). In addition, the overlapped proteins with the training set were excluded. As a result, we obtained a testing dataset containing 755 protein chains with lengths ranging from 50 to 1000 residues.

#### Calculation of solvent accessibility

In our study, solvent accessibility was calculated using DSSP [27], and relative solvent accessibility (RSA) was calculated by dividing solvent accessibility by the maximum solvent accessibility [2]. The calculated RSA was further divided into two states, namely buried state and exposed state, using the exposure threshold of 0.25 [14].

### Analysis of prediction performance

#### Comparison of prediction performance with state-of-the-art approaches

**Fig. 2:**
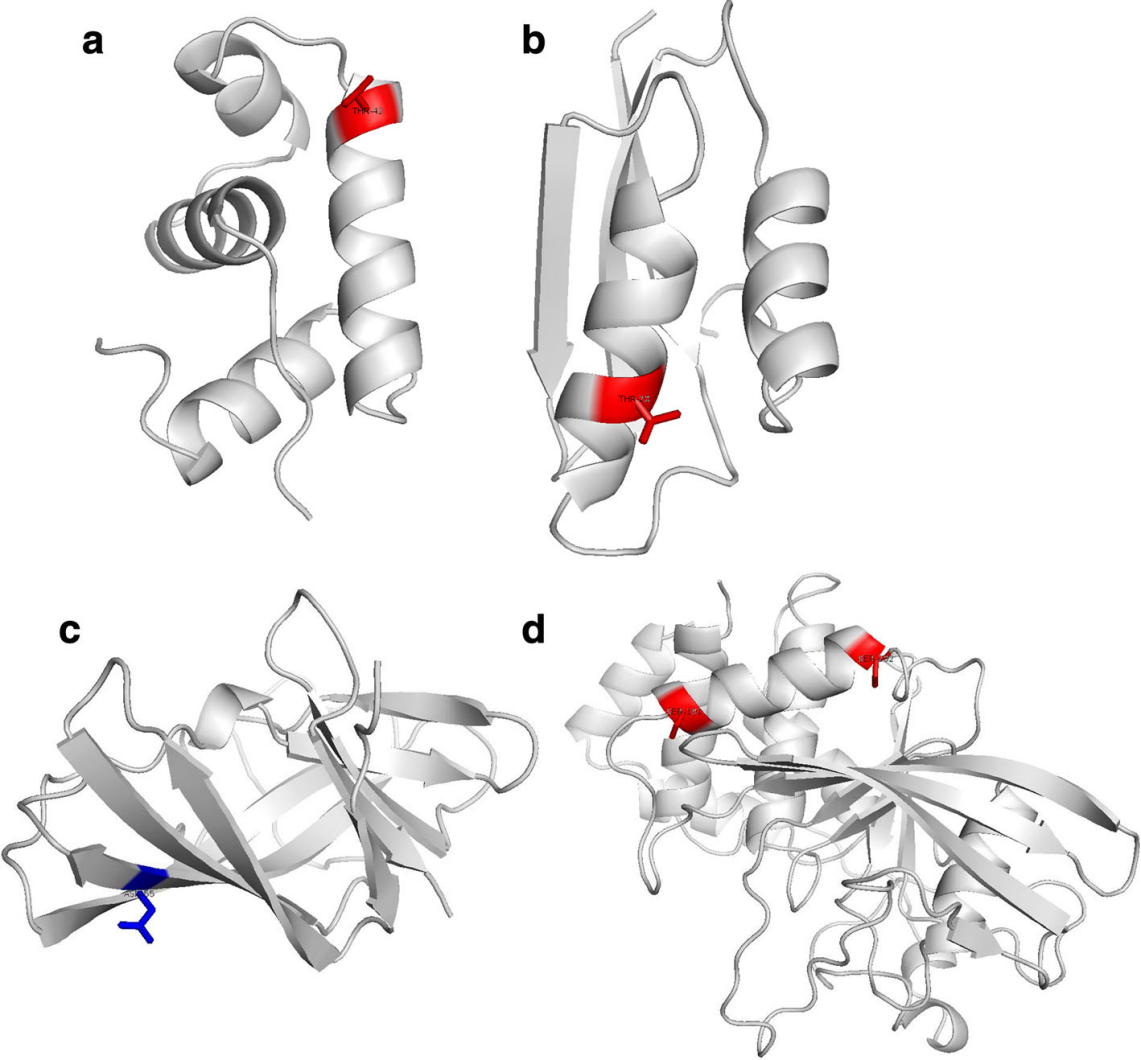
Case studies of the predicted results for protein 1fse (**a**), 1osd (**b**), 1lmi (**c**), and 1l8k (**d**). Here, exposed residues are colored in *red*, whereas buried residues are colored in *blue*. For these residues, ACRF correctly predicted their burial states, while BBRN failed. **a** Protein 1fse in *α* dataset. **b** Protein 1osd in *α*+*β* dataset. **c** Protein 1lmi in *β* dataset. **d** Protein 1l8k in *α*/*β* dataset

We compared the high-order CRF model with the widely-used BRNN model. For the sake of fair comparison, we fed these two models with identical features as input, trained them on the same training set, and evaluated them based on the same validation set. As shown in Table 1, the accuracies of *ACRF* are 0.8, 0.8, 0.6, and 1.0% higher in the four datasets *α*,*β*,*α*/*β*, and *α*+*β*, respectively, when compared with the BRNN model. As concrete examples, Fig. 2 shows four proteins with residues incorrectly predicted by the BRNN model but correctly predicted by ACRF. These results showed that ACRF had better performance than BRNN when identical features were used. In addition, ACRF also outperforms the logistic regression model, suggesting the importance of incorporating correlations into the prediction model.

**Table 1:** Prediction accuracy of ACRF and BRNN on the four datasets *α*,*β*,*α*/*β*,*α*+*β*

Methods	*α*	*β*	*α*/*β*	*α*+*β*
*LR*	0.821 ±0.005	0.801 ±0.004	0.808 ±0.003	0.809 ±0.005
*BRNN*	0.825 ±0.004	0.805 ±0.003	0.812 ±0.004	0.812 ±0.006
*ACRF*	0.833 ±0.006	0.813 ±0.005	0.818 ±0.003	0.822 ±0.005
*ACRF-CN*	0.806 ±0.006	0.785 ±0.004	0.787 ±0.003	0.794 ±0.006
*ACRF-CN-SC*	0.805 ±0.004	0.782 ±0.005	0.783 ±0.005	0.789 ±0.007
*ACRF-CN-SC-SS*	0.801 ±0.004	0.769 ±0.004	0.773 ±0.005	0.784 ±0.005

For the sake of fair comparison, *ACRF* and *BRNN* use identical feature sets. To investigate the effects of different features on prediction accuracy, we evaluated a set of variants of ACRF, including *ACRF-CN* with contact number removed, *ACRF-CN-SC* with both contact number and sequence conservation removed, and *ACRF-CN-SC-SS* with contact number, sequence conservation, and secondary structure information removed from the ACRF model

Besides the BRNN model, we also compared ACRF only with the newly-released proteins using the state-of-the-art prediction tool ACCpro on the testing dataset. On this testing dataset, the prediction accuracies of these two tools are 0.768 and 0.765, respectively. When limited to short proteins with less than 300 residues, the prediction accuracies are 0.769 and 0.760, respectively. In addition, the logistic regression model shows a prediction accuracy of 0.755. These results suggest that ACRF has the best performance in RSA prediction, particularly for proteins with shorter sequences.

### Analyses of the effects of features on prediction accuracy

As the ACRF model consists of a variety of features, it is interesting to investigate the effects of different features, including residue types, SS, sequence conservation (SC), contact numbers (CN), and high order terms, on prediction accuracy. Therefore, we evaluated ACRF without the SS, SC, and CN features. The effects of these features are summarized as below.

#### Effects of residue types

**Fig. 3:**
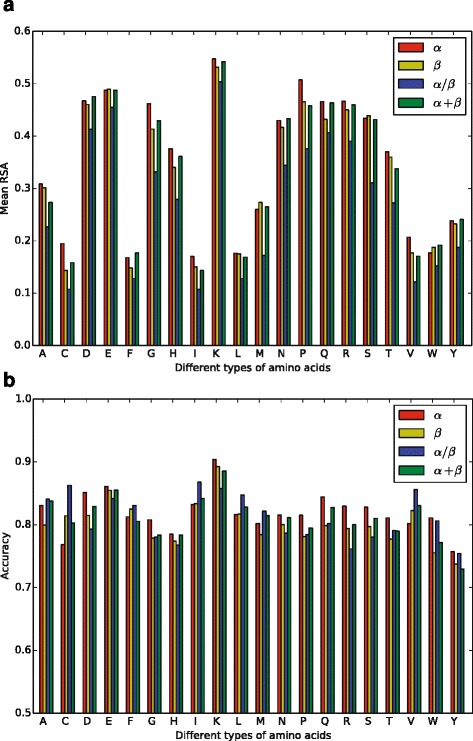
Effects of residue types for prediction accuracy of RSA. Panel (**a**) shows that RSA is closely related with residue types. In panel (**b**), the prediction accuracy of different residue types is shown. **a** Relationship between average RSA and residue type. **b** Prediction accuracy of residues with different types

As shown in Fig. 3 a, the RSA of residues is tightly related to residue types. Specifically, residues C, F, I, L, and W show low average RSA, whereas D, E, K, Q, and R show high average RSA. This observation can be explained according to the physical-chemical properties of residues, i.e., F, I, L, W have high hydrophobicity and C usually forms disulfide bonds; in contrast, D, E, K, Q, R are either charged or polar and thus tend to be exposed.

It should be noticed that for the residue Y, the prediction accuracy is usually low. A reasonable explanation might be the special structure of Y — it has a hydrophobic benzene ring but a polar hydroxyl group on the benzene ring. This special structure leads to various RSAs of Y in different proteins. In addition, although residue C usually shows significant preference for low average RSA, the average RSA of C is close to the exposure threshold 0.25 in the *α* dataset, making it difficult to predict.

#### Effects of secondary structures

**Fig. 4:**
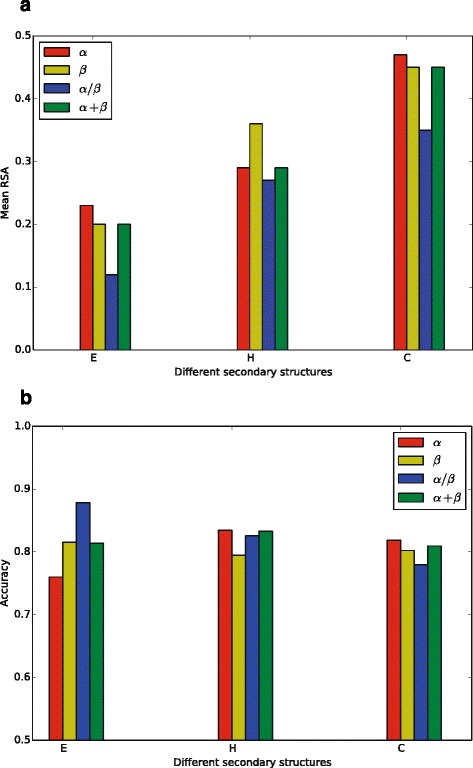
Effects of secondary structural information on prediction accuracy of RSA. Panel (**a**) shows the strong correlation between average RSA and SS type of residues, while panel (**b**) shows the prediction accuracy for residues with different SS types. **a** Average RSA of residues with different SS types. **b** Prediction accuracy of RSA for residues with different SS types

Figure 4 a suggests that *β*-strands tend to be buried, coils tend to be exposed, and *α*-helices tend to be half-buried and half-exposed. This tendency implies that the incorporation of SS information in prediction model should facilitate the prediction of RSA. To investigate the effect of SS information, we evaluated two variants of ACRF, namely, *ACRF-CN-SC* with SS taken into consideration and *ACRF-CN-SC-SS* with SS features removed from the model. As shown in Table 1, the prediction accuracies of *ACRF-CN-SC* are 0.4, 1.3, 1.0, and 0.5% higher than that of *ACRF-CN-SC-SS* in the four datasets, respectively.

However, the effects of SS information on prediction accuracy change with protein types. For *β* strands, ACRF achieves a prediction accuracy of 86% in the *α*/*β* dataset and 76% in the *α* dataset. Similarly, for coils, the prediction accuracy is 82% in the *α* dataset, which is higher than the accuracy of 78% in the *α*/*β* dataset (Fig. 4 b).

#### Effects of sequence conservation and contact number

**Fig. 5:**
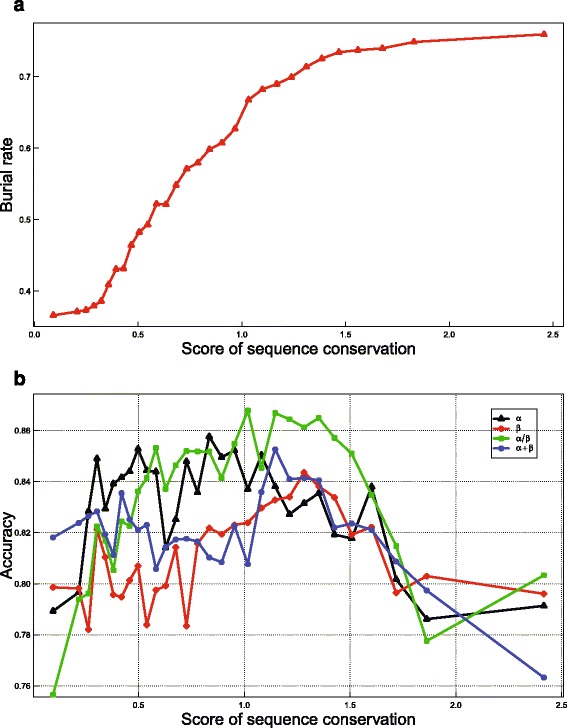
Effects of sequence conservation information on prediction accuracy of RSA. Panel (**a**) shows that as sequence conservation increases, the ratio of buried residues increases, too. Panel (**b**) suggests that the prediction accuracy reaches its maximum for residues with intermediate sequence conservation. **a** Relationship between sequence conservation and the ratio of buried residues. **b** Relationship between prediction accuracy and sequence conservation

**Fig. 6:**
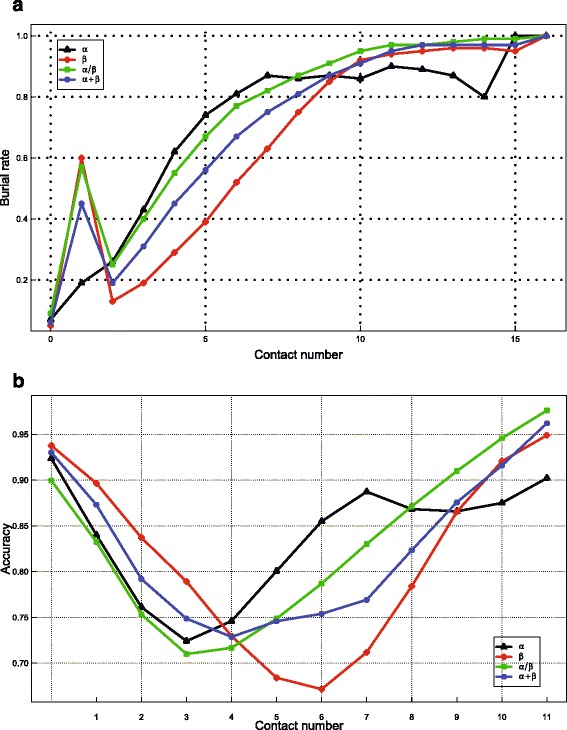
Effects of contact number information on prediction accuracy of RSA. Panel (**a**) shows that as contact number increases, the ratio of buried residues increases, too. Panel (**b**) suggests that the prediction accuracy reaches its minimum for residues with intermediate contact number. **a** Relationship between contact number and the ratio of buried residues. **b** Relationship between contact number and prediction accuracy

Figure 5 a shows a strong correlation between RSA and SC of residues. Similarly, strong correlations were also observed between RSA and CN of residues (Fig. 6 a). Thus, the incorporation of SC and CN should facilitate the prediction of RSA. To investigate the effects of these two types of features, we compared ACRF with two of its variants, namely, *ACRF-CN* with CN features removed and *ACRF-CN-SC* with both SC and CN features removed. As shown in Table 1, the prediction accuracies of *ACRF* are 2.7, 2.8, 3.1, and 2.8% higher than that of *ACRF-CN*, and the prediction accuracies of *ACRF-CN* are 0.1, 0.3, 0.4, and 0.5% higher than that of *ACRF-CN-SC* in the four datasets, respectively. These results clearly suggest the importance of incorporating these two types of features in prediction.

Interestingly, Fig. 5 b shows that for residues with too high or too low SC, the prediction accuracy is usually low, whereas for residues with medium SC, the prediction accuracy reaches its maximum. In contrast, ACRF shows higher prediction accuracy for residues with significantly larger or smaller CN (Fig. 6 b).

## Conclusion

In this study, we present a high-order CRF model for predicting the burial states of protein residues. The novelty of the model is that it can explicitly explore the correlation of burial states among residues. In addition, a variety of features, including SC and CN, were incorporated into the model. Experimental results on two benchmark datasets show that our approach outperforms the logistic regression approach and state-of-the-art neural network model. This will greatly facilitate the studies on protein structure, evolution, and functions.

## Method

In this section, we first describe the high-order CRF model with an emphasis on the feature terms to represent correlations. Then we present the procedures for parameter training and inferring, followed by features used in this model.

### High-order CRF model

CRF is a widely-used discriminant model for classification [28]. One of the CRF models, chain CRF, uses singlet and one-order doublet feature functions only; thus, chain CRF is can only consider the correlation among adjacent residues. In order to describe the correlation among long-distance residues, we added high-order terms into the chain CRF model to construct a high-order CRF model. More specifically, a four-order term was designed to describe the correlation among residue pairs *A*_*i*_−*A*_*i*+4_ and *A*_*i*_−*A*_*i*+3_ on *α*-helices, and a two-order term was designed to capture the correlation among residue pairs *A*_*i*_−*A*_*i*+2_ on *β*-strands. Here, *A*_*i*_−*A*_*i*+*d*_ denotes a pair of residues with a sequence separation of *d* amino acids. Since on coil regions, no strong correlation among long-range residue pairs was observed, a one-order term is sufficient for describing the correlation among adjacent residues.

**Fig. 7:**
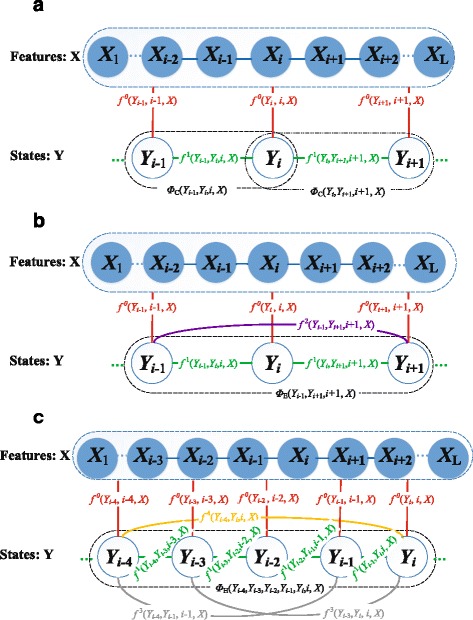
High-order CRF model for prediction of burial states of residues. The model consists of four-order term for *α* helices (panel **a**), two-order terms for *β* strands (panel **b**), and one-order terms for coils (panel **c**). Here, solid points denotes features of the model, hollow points indicate solvent accessibility, and *f* ^4^,*f* ^3^,*f* ^2^,*f* ^1^,*f* ^0^ are four-order, three-order, two-order, one-order doublet feature functions and singlet feature functions, respectively. **a** One-order CRF for residues in coils. **b** Two-order CRF for residues on *β*-strands. **c** Four-order CRF for residues on *α*-helices

The high-order CRF model is graphically shown in Fig. 7. Specifically, for a protein sequence with a length of *L*, we use *Y*=*Y*_1_ *Y*_2_...*Y*_*L*_ to denote the sequence of burial states and *X* to denote the feature sets. The high-order CRF model is described as below.

$${{\begin{aligned} {}p(Y|X)&=p(Y_{1},...,Y_{L}|X) = \frac{1}{Z(X)} \prod_{j=1}^{n} \left(g_{h} + g_{s} + g_{c} \right) \\[-4pt] g_{h}&=I(e_{j}=H) \prod_{i=s(e_{j})+4}^{t(e_{j})} \!\exp \left(\phi_{H}(Y_{i-4}, Y_{i-3}, Y_{i-2}, Y_{i-1}, Y_{i}, i, X) \right)  \\[-4pt] g_{s}&=I(e_{j}=E) \prod_{i=s(e_{j})+2}^{t(e_{j})} \exp \left(\phi_{E}(Y_{i-2}, Y_{i-1}, Y_{i}, i, X) \right)  \\[-4pt] g_{c}&=I(e_{j}=C) \prod_{i=s(e_{j})+1}^{t(e_{j})} \exp \left(\phi_{C}(Y_{i-1}, Y_{i}, i, X) \right)  \end{aligned}}} $$

Here, *Z*(*X*) denotes the partition function for normalization, *n* denotes the number of SS segments of the target protein sequence, and *e*_*j*_ denotes the *j*-th SS segment with *s*(*e*_*j*_) denoting the start position and *t*(*e*_*j*_) denoting the end position. *I*(*e*_*j*_=*H*),*I*(*e*_*j*_=*E*), and *I*(*e*_*j*_=*C*) are index functions, which take 1 if the corresponding conditions hold and 0 otherwise. The terms *ϕ*_*H*_(*Y*_*i*−4_,*Y*_*i*−3_,*Y*_*i*−2_,*Y*_*i*−1_,*Y*_*i*_,*i, X*),*ϕ*_*E*_(*Y*_*i*−2_,*Y*_*i*−1_,*Y*_*i*_,*i, X*), and *ϕ*_*C*_(*Y*_*i*−1_,*Y*_*i*_,*i, X*) were introduced to describe the correlations among continuous residues. These terms are formally described as below:

$$ {{\begin{aligned} \phi_{H}(Y_{i-4}, \!Y_{i-3}, \!Y_{i-2}, \!Y_{i-1}, \!Y_{i}, \!i, \!X) &\,=\,\!\sum_{j}{\theta_{j}\, f^{0}_{j}(Y_{i}, i, X)} \,+\,\! \sum_{j}{\lambda_{j}\, f^{1}_{j}(\!Y_{i-1},\!Y_{i}, i, X)}  \\ &+\! \sum_{j}\!{\gamma_{j}\, f^{3}_{j}(Y_{i-3},\!Y_{i}, \!i, X)} \,+\,\! \sum_{j}{\!\tau_{j}\, f^{4}_{j}\!(Y_{i-4},\!Y_{i}, \!i, \!X)}  \end{aligned}}}  $$

$${{\begin{aligned} \phi_{E}(Y_{i-2}, Y_{i-1}, Y_{i}, i, X)\!&=\!\sum_{j}{\theta_{j}\, f^{0}_{j}(Y_{i}, i, X)} + \sum_{j}{\lambda_{j} \, f^{1}_{j}(Y_{i-1},Y_{i}, i, X)} \\ &+ \sum_{j}{\mu_{j}\, f^{2}_{j}(Y_{i-2},Y_{i}, i, X)} \end{aligned}}} $$

$$ \phi_{C}(Y_{i-1}, Y_{i}, i, X)\,=\,\sum_{j}{\theta_{j}\, f^{0}_{j}(Y_{i}, i, X)} + \sum_{j}{\lambda_{j}\, f^{1}_{j}(Y_{i-1},\!Y_{i}, i, X)} $$

where $f^{0}_{j}(Y_{i}, i, X)$ is the singlet function, and $f^{1}_{j}(Y_{i-1},$ $Y_{i}, i, X), f^{2}_{j}(Y_{i-2},Y_{i}, i, X), f^{3}_{j}(Y_{i-3},\!Y_{i}, i, X), f^{4}_{j}(Y_{i-4},$ *Y*_*i*_,*i, X*) are the one-order, two-order, three-order, and four-order doublet functions, respectively. Here, *Λ*=(*θ*,*λ*,*μ*,*γ*,*τ*) denotes the weights of these singlet and doublet terms.

### Parameter estimation

In this study, gradient descend technique was employed for parameter estimation to maximize the following likelihood:

1 $$\begin{array}{@{}rcl@{}} \mathcal{L}_{\Lambda}&=&log \prod_{m} {p(Y^{m},X^{m})} \end{array} $$

where (*X* ^*m*^,*Y* ^*m*^) denote the *m*-th protein in the training set. *X* ^*m*^ consists of the feature set, which is the input to the model, and *Y* ^*m*^ denotes the calculated burial state labels.

It should be noticed that the calculation of gradient depends on the partition function *Z*(*X* ^*m*^); however, the direct computation of *Z*(*X* ^*m*^) takes exponential time. Here, we employed the forward-backward technique [29] to efficiently calculate the partition function.

### Inferring procedure

In this study, the marginal probability is maximized for inferring burial states of residues. In *α*-helices, *β*-strands and coils, the marginal probability *p*(*Y*_*i*_=*y*_0_|*X*) of the *i*-th residue is calculated with corresponding forward vectors and backward vectors. Let *α*_*c*_,*β*_*c*_,*α*_*e*_,*β*_*e*_,*α*_*h*_, and *β*_*h*_ indicate the logarithm vectors of forward factors and backward factors on *α*-helices, *β*-strands, and coils, respectively. In addition, *Z*(*X*) indicates the logarithm of the partition function. The burial state *y*_*i*_ is predicted as below.

2 $$\begin{array}{@{}rcl@{}} y_{i}^{*}={argmax}_{y_{0}} p(Y_{i}=y_{0}|X) \end{array} $$

The conditional probability *p*(*Y*_*i*_=*y*_0_|*X*) is calculated according to the SS type of the *i*-th residue as follows.

If the *i*-th residue is in coil region, we have

3 $$\begin{array}{@{}rcl@{}} p(y_{0}|X)=&\sum_{y_{1}} \exp \{ \alpha_{c}(y_{1}, i-1) + \beta_{c}(y_{0}, i) - Z(X)  \\ &-{ \theta^{T}} f^{0}(y_{0}, i, X) - { \lambda^{T}} f^{1}(y_{1}, y_{0}, i, X) \} \end{array} $$

If the *i*-th residue is in *β*-strands, we have

4 $$ \begin{aligned} p(y_{0}|X)=&{\sum\nolimits}_{y_{1}} {\sum\nolimits}_{y_{2}} \exp \{ \alpha_{e}(y_{2}, y_{0}, i-1) +\beta_{e}(y_{1}, y_{0}, i) \\ &+ { \mu^{T}} f^{2}(y_{2}, y_{0}, i, X)\,-\,Z(X)-{ \theta^{T}} f^{0} (y_{1}, i-1, X) \} \end{aligned}  $$

If the *i*-th residue is in *α*-helices, we have

5 $$ \begin{aligned} p(y_{0}|X)\,=\,&{\sum\nolimits}_{y_{1}} {\sum\nolimits}_{y_{2}} {\sum\nolimits}_{y_{3}} {\sum\nolimits}_{y_{4}} \exp \{ \alpha_{h}(y_{4}, y_{3}, y_{2}, y_{1}, i-1) \\ &+\beta_{h}(y_{3}, y_{2}, y_{1}, y_{0}, i-3) - { \theta^{T}} (f^{0} (y_{3}, i-3, X) \\ &+f^{0} (y_{2}, i-2, X) + f^{0} (y_{1}, i-1, X)) - Z(X) \\ &-{ \lambda^{T}} (f^{1} (y_{3}, y_{2}, i-2, X) + f^{1} (y_{2}, y_{1}, i-1, X)) \} \end{aligned}  $$

### Features

Our high-order CRF model consists of two types of features, namely, singlet features and doublet features.

#### Singlet features

Here, a total of 119×*N* singlet features were used, where *N*=2 denotes the number of burial states.

- Amino acid-related features: These features include residue types, sequence distance to the residue of interest [10], N terminal and C terminal residues [30], tendency related to the physicochemical properties [10, 30, 31], probabilities of being disordered [10], and probabilities of being a binding site [10].
- SC features: We used the sequence profile generated by running PSI-BLAST [32] with three iterations and E-value 0.001 (20×*N* features). In addition, SC of each residue was calculated by comparing the sequence profile against background distribution [33].
- Structural features: We used the predicted SS information reported by PSIPRED [34] and DeepCNF [35], the end tendency of SS [36] (11×*N* features), and the I-site score [37] (1×*N* features).
- CN information: These features include CN predicted using AcconPred [2] (1×*N* features), and contact potentials of position pairs [38] (40×*N* features). For a certain residue, its CN denotes the number of residues with spatial distance less than 8 Å and sequence separation of at least 5 amino acids.

#### Doublet features

A total of nine doublet features were used, including three four-order features, three three-order features, and three two-order features. Among them, four-order features and three-order features are used on *α*-helices and two-order features are used on *β*-strands. Each doublet feature consists of mutual information, *cosine* similarity, and contact map [39] calculated based on sequence profiles.

## Abbreviations

BRNN: Bidirectional recurrent neural network
CRF: Conditional random field
RSA: Relative solvent area
